# Effect of magnetic field on improvement of photocatalytic performance of V_2_O_5_/TiO_2_ nanoheterostructure films prepared by sparking method

**DOI:** 10.1038/s41598-022-05015-2

**Published:** 2022-02-10

**Authors:** Porntipa Pooseekheaw, Winai Thongpan, Ekkapong Kantarak, Wattikon sroila, Tewasin Kumpika, Pisith Singjai

**Affiliations:** 1 Department of Physics and Materials Science, Faculty of Science, Chiang Mai University, Chiang Mai, 50200 Thailand; 2 PhD’s Degree Program in Applied Physics, Faculty of Science, Chiang Mai University, Chiang Mai, 50200 Thailand; 3 Center of Excellence in Materials Science and Technology, Chiang Mai University, Chiang Mai, 50200 Thailand

**Keywords:** Materials science, Nanoscience and technology, Optics and photonics

## Abstract

Our porous V_2_O_5_/TiO_2_ nanoheterostructure films (with a Ti/V atomic ratio of 1:1) were fabricated via a single-step sparking method using a strong magnetic field (0.5 T) without annealing requirement for the first time. We found that the magnetic flux arrangement has effect on film crystallization, unique morphology, large specific surface area, and surprisingly controllable phase structure of the films. An amorphous TV film was transformed to the TiO_2_ (anatase/rutile) phase (for TVN) and V_2_O_5_–VO_2_ mixed-phase (for TVH) without destroying the mesopores from an annealing process. Moreover, the TVH sample able to improve the degradation rate up to 270% compared with pre-annealed TV films and up to 30% with post-annealed (400 °C) TVA films. In this paper, the influence of magnetic flux arrangement on structural, morphological, optical, and photocatalytic properties of prepared sample have been investigated and reported.

## Introduction

Titanium dioxide (TiO_2_) has been widely investigated and has commercial applications in several fields (self-cleaning applications, water and air purification, and as a water-splitting catalyst) because of its photostability, non-toxicity, cost-effectiveness, and excellent degradation capacity^1–3^. However, a large band gap (3.2 eV) and low quantum yields of TiO_2_ have limited its practical applications since it can be only activated by the illumination of UV light (5% of the solar spectrum). Therefore, considerable efforts have been devoted to TiO_2_ for facilitating its visible light absorption.

Photocatalytic performance is well-known that can be effectively improved through the designs of large surface morphologies and the interface heterojunctions of composite films^4–6^. In the literature review, we found that heterostructures of TiO_2_ and other oxides, such as Cu_2_O, WO_3_, V_2_O_5_, ZnO, SiO_2_, MoS_2_, Fe_3_O_4_ and SnO_2_^7–11^ to form the semiconductor coupling are believed to overcome the facile recombination of e^−^/h^+^ pairs, which is a promising method to optimize photocatalytic performance and visible-light utilization. In our previous work, porous vanadium pentoxide/titanium dioxide (V_2_O_5_/TiO_2_) nanoheterostructure films with various atomic ratios of Ti/V (4:1, 2:1, 1:1, and 1:2) were successfully fabricated by the sparking method which can synthesize highly porous and composite films in one simple step^12^. However, post-annealed process was required for films crystallization, oxidation, and strong interaction between nanoparticles that limits the application for large-scale manufacturing with simplicity and cost-effectiveness. In addition, film’s mesopores was destroyed by agglomeration of particle in the annealing process^13,14^. The novel design to improve the sparking method need immediate further study.

Normally, nanoparticles released off the metal tips are electrically charged, then moves under the electric fields generated by the metal wires. Therefore, the films are deposited on the surface only in the direction of the electric flux ($\stackrel{\rightharpoonup}{E}$) parallel to the plane direction of the surface ($\stackrel{\rightharpoonup}{A}$) relative to the dot product vector ($deposition pattern=\stackrel{\rightharpoonup}{E}.\stackrel{\rightharpoonup}{A}$). That is challenging to control the direction of nanoparticles to improve deposition rate, uniformity, and morphology of prepared films. In previous paper, an external electric field was applied to confine nanoparticles on the substrate for increase deposition rate and uniformity of the films^15^. However, this technique is only suitable for conductive substrates (insulating substrates are not recommended). In this paper, we focused on magnetic fields effect that is highly promising to increase deposition rate and improve film alignment. Moreover, it also promotes film crystallization as a substitute of annealing process.

The charged particle (q) traveling through a magnetic field are exposed to the Lorentz Force, which is the interaction of the applied magnetic field and the magnetic field generated by moving particles^16^. This phenomenon is directly related to velocity (v), the strength of the applied magnetic field (B), and their angle ($\theta$), which magnitude of force is determined by $\stackrel{\rightharpoonup}{F}=qvBsin\theta$. 

Therefore, this present work provides an alternative route for fabrication of porous V_2_O_5_/TiO_2_ nanoheterostructure films prepared by the sparking method under high magnetic field without annealing process. The influence of magnetic flux arrangement on structural, morphological, optical, and photocatalytic properties of prepared sample have been investigated and reported in this work.

## Methods

### Nanocomposite synthesis

We fabricated the porous V_2_O_5_/TiO_2_ nanocomposite films on quartz glass substrates for 60 min in an atmospheric with a single process by sparking method using high magnetic field at 0.5 T. The experiment was performed by sparking off titanium wires (Ti, ø 0.25 mm, purity 99.5%, Advent Research Material Ltd.) and vanadium wires (V, ø 0.25 mm, purity 99.8%, Advent Research Material Ltd.) with different input DC voltages of 5 V (I = 0.5 A) and 3.5 V (I = 0.4 A), respectively. The nanoparticle was sparking off the wire with difference quantities by a breakdown voltage of ~ 3 kV that was discharged from 24 nF capacitor. These conditions were fixed throughout this work to synthesize porous V_2_O_5_/TiO_2_ nanocomposite films with a V/Ti atomic ratio of approximately 1:1, which was also the optimized condition for improving photocatalytic performance reported in previous paper^12^.

**Figure 1:**
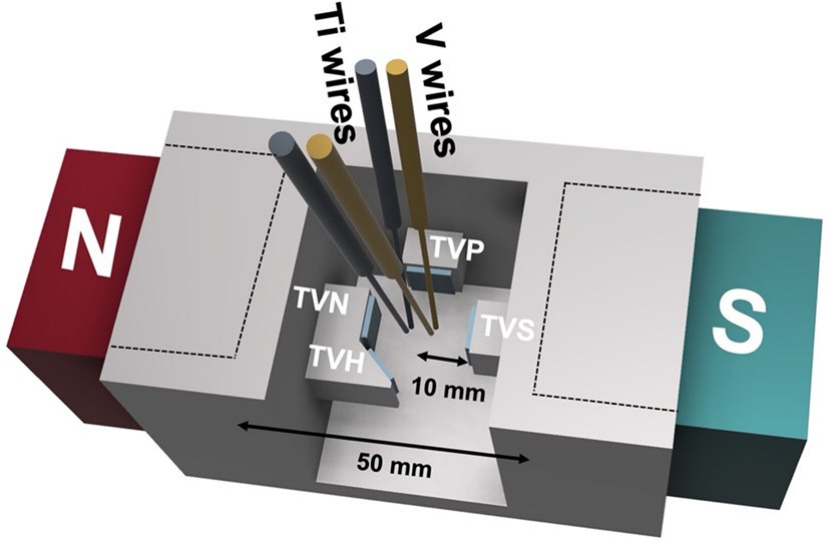
Schematic diagram of the sparking method under various magnetic flux arrangements.

The influence of magnetic flux arrangement on the sample was investigated by locating the substrate on north, south, parallel, and 45 degrees of the magnetic poles, which were labeled as TVN, TVS, TVP, and TVH respectively, as shown in Fig. 1. And compared with non-magnetic field for pre- and post-annealed at 400 °C for 1 h at atmospheric pressure, which was labeled as TV and TVA, respectively.

### Characterization techniques

The morphologies of films were characterized by FE-SEM (JEOL JSM 6335F). The Raman spectra were recorded using JOBIN YVON HORIBA T64000 with a 514 nm of an Ar laser. The oxidation states and chemical composition of elements were analyzed by XPS (AXIS Ultra DLD, Kratos Analytical Ltd.). The photocatalytic reaction of samples was evaluated by 0.01 mmol/L methylene blue (MB) solution (Ajex Finechem) under visible light was produced by a 75 W Xenon lamp with light intensity ~ 100 w/m^2^ (Philip) for 1–5 h. The optical properties and variations in the concentration of MB with samples were analyzed at given irradiation times by a UV–Vis spectrophotometer (Varian Cary 50).

## Results and discussions

### Surface morphological

**Figure 2:**
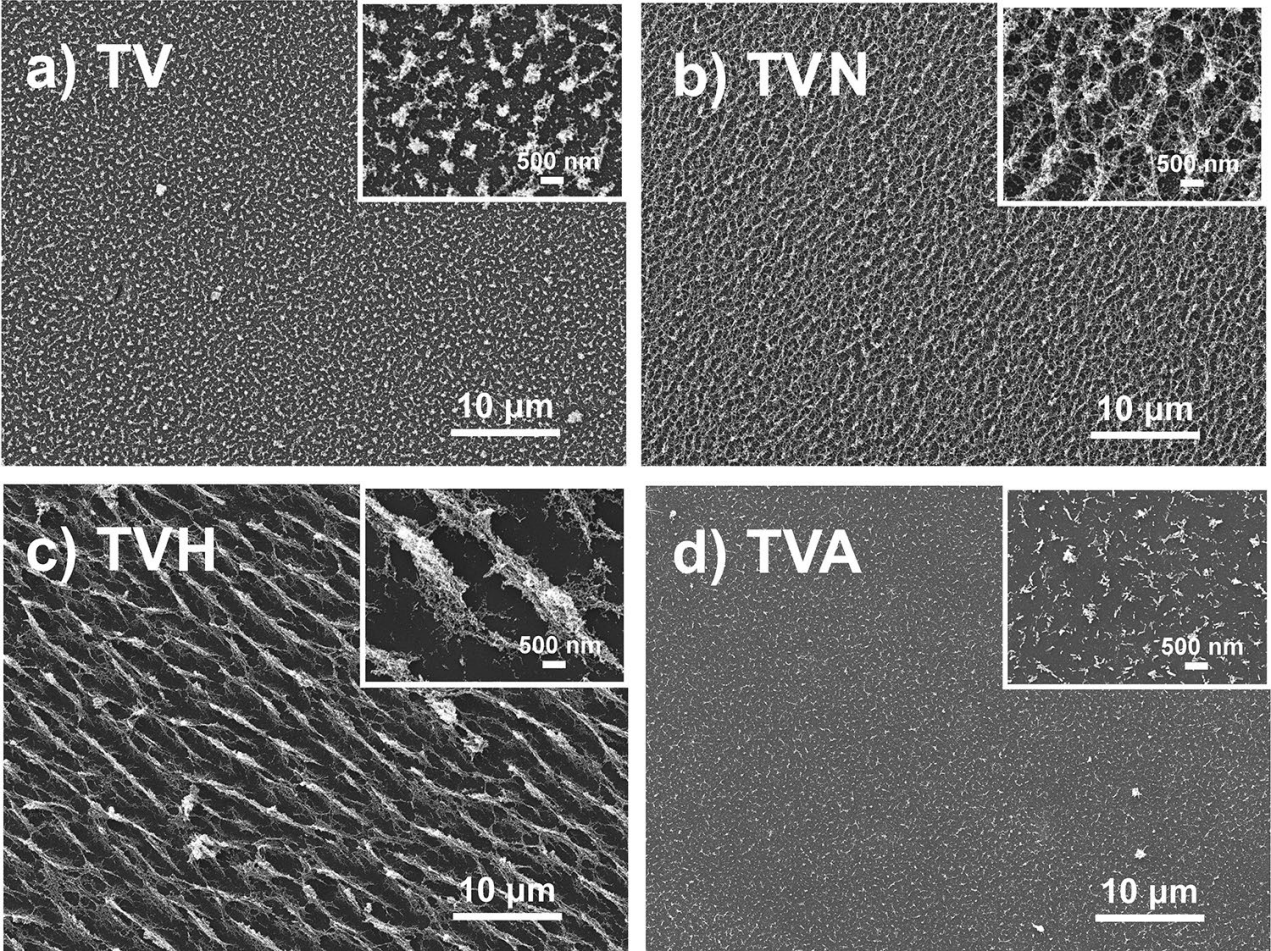
Surface morphology of samples located in different magnetic fluxes arrangements.

The porosity structure of films, which was the highlight of our sparking method, regularly collapsed during a high-temperature annealing process due to the film crystallization^13,14^, as shown in the TVA sample (Fig. 2d). In this work, a unique arrangements of particle morphology clearly illustrated after exposure to the magnetic field observed in FE-SEM images. The traditional porous morphology on TV sample (Fig. 2a) reformed to superior fluffy morphology for TVN sample and arrangement in a slope line for TVH sample as shown in Fig. 2b,c, respectively. This phenomenon is explained by the movement of synthesized particles affected by the Lorentz Force. Normally, magnetic forces can force charged particles to move in circular motion when they travel perpendicular to the magnetic field. On the other hand, other particles traveling at their velocity at some arbitrary angle with respect to magnetic field lines, its path is a helix, which circular radius is given by $r=\frac{mv(sin\theta )}{qB}$. Therefore, at a narrow angle, the nanoparticles move in small helix paths. This produces a uniform and non-pattern morphology as shown in the TVN sample. In the case of the wide angle TVH sample, large-scale spiral motion of the particles is generated resulting in a clear orientation pattern of the film. These modified morphologies significantly increase the specific surface area, which is largely beneficial for the adsorption of dye molecules and promotes photo-generated charge transport on the surface of films^17^.

### Raman spectroscopy

**Figure 3:**
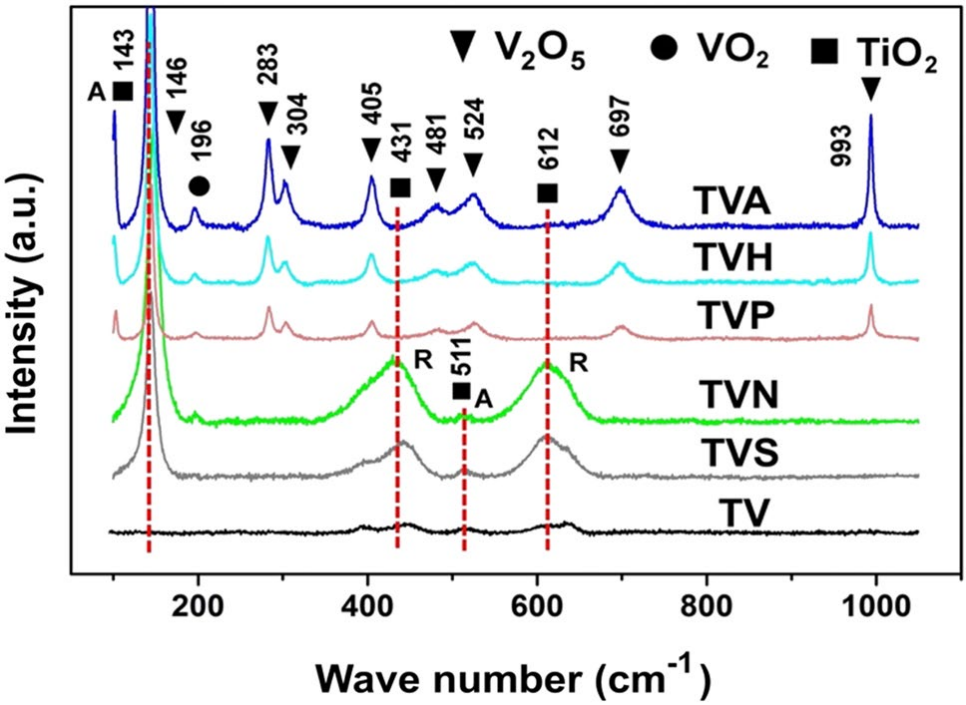
Raman spectra of prepared samples.

Figure 3 illustrates the Raman spectra of various crystalline phases of V_2_O_5_/TiO_2_ nanoheterostructure samples. These results were the first to be noticed and surprising. First, the amorphous structure was observed in the TV sample through the absence of peaks^18^. Second, phase structure turned to great TiO_2_ (anatase/rutile) phase for TVN and TVS sample, normally occurred after post-annealed over 400 °C as discussed in previous paper^19,20^. Third, the TVH and TVP sample provided eight strong peaks of lattice vibrations of orthorhombic V_2_O_5_ and one weak peak of VO_2_ indicating the V_2_O_5_-VO_2_ modification phase^21,22^ as also correlating with post-annealed sample (TVA). This result confirmed that the phase structure of the films was altered and reformed by located flux arrayment of magnetic field without the annealing. There are reported that the temperature or magnetic field could control on the spins of dangling bonds polarization^23^. Therefore, we presumed that the applied magnetic field causes the phase transform from amorphous to crystalline film. Moreover, it retains the morphology of the porous film, which provide the efficiency of surface reactions significantly support photocatalytic activities.

### X-ray photoelectron spectroscopy

**Figure 4:**
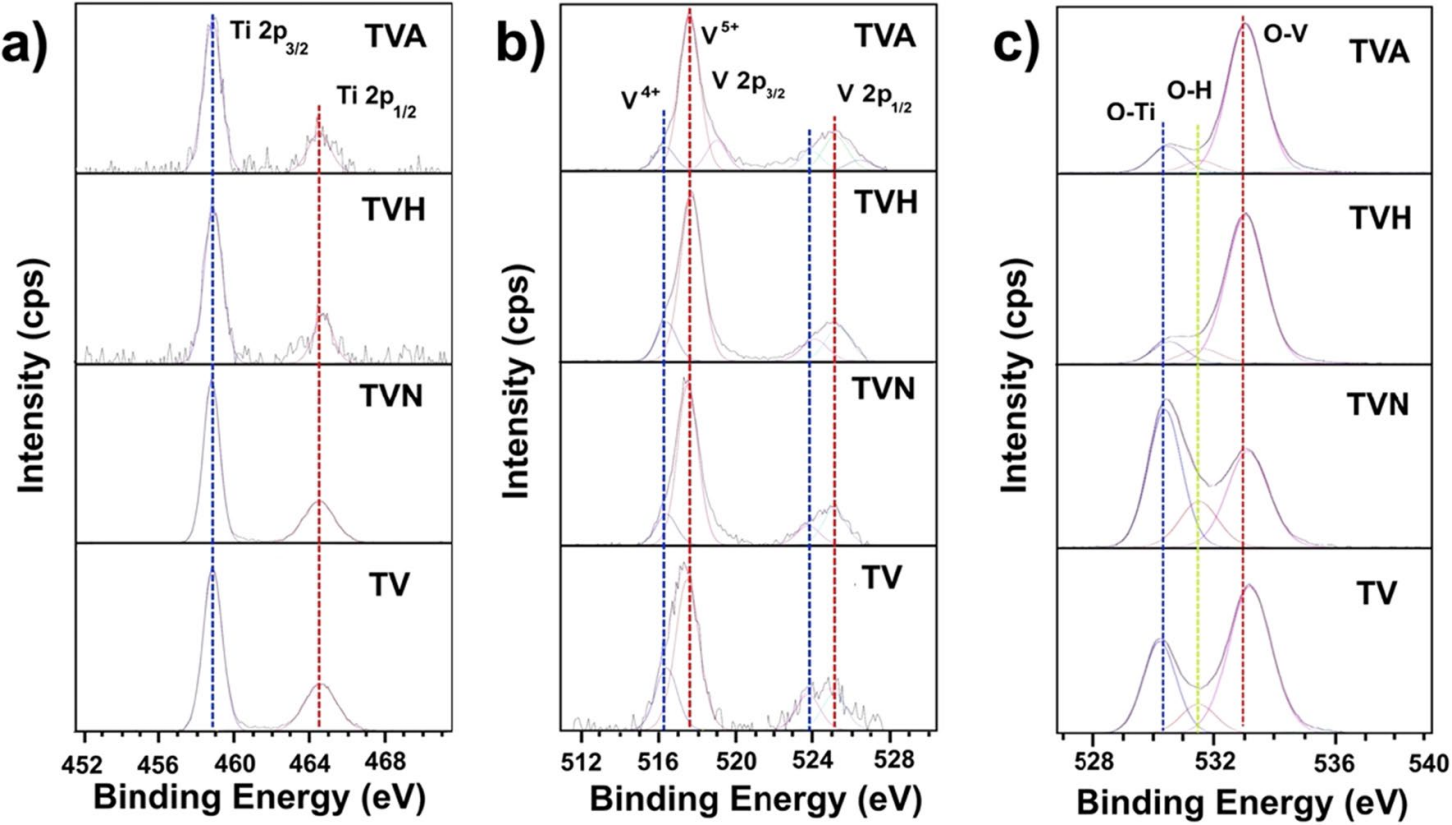
High-resolution XPS spectra of (**a**) Ti 2p, (**b**) V 2p, and (**c**) O 1s for prepared samples.

The comparison of high-resolution XPS spectra for Ti 2p, V 2p, and O 1s of prepared films with various conditions as illustrated in Fig. 4a–c. For Ti 2p state, the located of Ti 2p_3/2_ and Ti 2p_1/2_ peaks, and their spin–orbit separation (5.6 eV), which is typical for Ti–O bonds in the TiO_2_ structure^24^. For V 2p state provide core level spectrum of V^5+^ and V^4+^ ions, which were centered at 517.5 and 516.2 eV, respectively, confirming that the V_2_O_5_/VO_2_ structure was formed and uniformly distributed on the prepared samples^25^. Figure 4c, three peaks at binding energies of 530.2 eV, 531.6 eV, and 533.0 eV observed in O 1s spectra also confirmed the O–Ti, O–H, and O–V bonding, respectively^12^. The area of O–Ti to O–V bond ratios on the TV, TVN, TVH, and TVA samples were approximately 0.50, 1.16, 0.13, and 0.15, respectively, indicating optimum integration of the TiO_2_ phase occurred in the TVN sample. Then it transfers to the V_2_O_5_–VO_2_ mixed-phase in the TVH sample, correlated with TVA sample, that a great consistent description of the Raman spectroscopy results.

The surfaces of nanostructures and amorphous phases always display the unpaired spins due to incomplete passivation of dangling bonds of surface ions^26^. Consequently, general nanoparticles immediately after releasing from the wires are probably contain many dangling bonds, which are easily polarized under the magnetic field^27^. Commonly, the spins of dangling bonds on the surface of nanoparticles are randomly oriented. A.V. Rodina and teams^23^ reported that an external magnetic field can control the spins of dangling bonds polarization, which spin polarization increases when a magnetic field is applied along direction of the spin-polarized electrons. As a result, the spins of unpolarized electrons are aligned along the magnetic field during deposition process. In other words, we report a method to manipulate electron spin polarization by designing magnetic flux alignment. Therefore, the difference in the magnetic flux alignment on the substrate of TVN and TVH samples are probably significant influence on phase transformation from amorphous to TiO_2_ (anatase/rutile) phase and V_2_O_5_–VO_2_ mixed-phase, respectively. However, the intrinsic mechanism behind these results is still unclear and rarely considered^28^.

### Optical and photocatalytic activity

**Figure 5:**
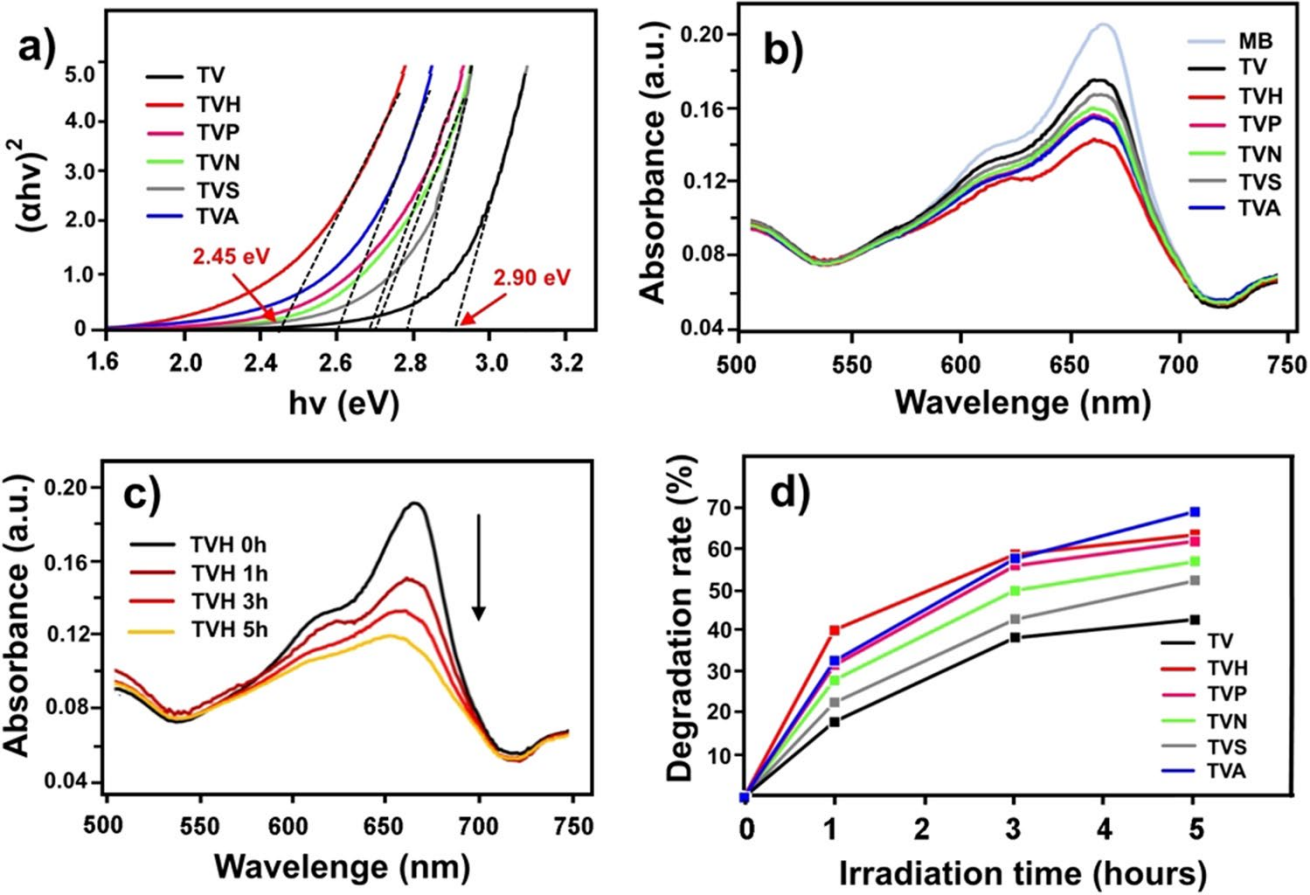
(**a**) Tauc plot, (**b**) absorption spectra for MB degradation in 1 h, (**c**) photocatalytic activity of TVH sample, and (**d**) percent photocatalytic degradation rate.

We found that the energy gap (E_g_) was modified by the strong magnetics field and the TVH sample presented a minimal value of E_g_ at 2.45 eV, indicating a large number of free electrons and a high amount of oxygen deficit in the sample^29^, as shown in Fig. 5a. The photocatalytic activity was evaluated through the MB degradation under visible-light irradiation for 1–5 h (Fig. 5b,c). From the results, all prepared films produced under magnetic field had prominent photocatalytic performances over the TV sample possibly due to their narrowed bandgap, re-crystallization, high porosity, and the strongly interface between TiO_2_ and V_2_O_5_ nanoparticles^30,31^. Unbelievable, the TVH sample provide great photocatalytic efficiency than post-annealed TVA sample. The TVH sample removed up to 41% of MB after 1 h of irradiation, which was a 270% and 30% increase compared to the without magnetic fields for pre- and post-annealed in the TV and TVA samples, respectively. However, the photocatalytic performance deteriorates over a long period activity time due to lack of strong films usually obtained by the annealing process, as can be seen in down trend of 5-h point in Fig. 5d.

## Conclusion

Superior porosity morphology, TiO_2_ (anatase/rutile)/V_2_O_5_–VO_2_ (mixed) phase controllable, and without annealing requirement of V_2_O_5_/TiO_2_ nanoheterostructure films were first successfully fabricated by a sparking method under a strong magnetic field and arrangement design. These films exhibit great chemical and physical properties (large absorbance adsorption abilities, high porosity morphology, strong interface heterojunctions, and low energy gap) that promoted excellent photocatalytic activity. We conclude that this novel technique could replace the high-temperature annealing process and retain the porous film morphology, that is a limitation of the annealing process, which opens a promising future for the films prepared by the sparking method without annealing.
